# Biocompatible Peritoneal Dialysis: The Target Is Still Way Off

**DOI:** 10.3389/fphys.2018.01853

**Published:** 2019-01-07

**Authors:** Maria Bartosova, Claus Peter Schmitt

**Affiliations:** Center for Pediatric and Adolescent Medicine Heidelberg, University of Heidelberg, Heidelberg, Germany

**Keywords:** peritoneum, peritoneal dialysis, glucose, glucose degradation products, biocompatibility, transformation, transport, junctions

## Abstract

Peritoneal dialysis (PD) is a cost-effective, home-based therapy for patients with end-stage renal disease achieving similar outcome as compared to hemodialysis. Still, a minority of patients only receive PD. To a significant extend, this discrepancy is explained by major limitations regarding PD efficiency and sustainability. Due to highly unphysiological composition of PD fluids, the peritoneal membrane undergoes rapid morphological and long-term functional alterations, which limit the treatment and contribute to adverse patient outcome. This review is focused on the peritoneal membrane ultrastructure and its transformation in patients with kidney disease and chronic PD, underlying molecular mechanisms, and potential systemic sequelae. Current knowledge on the impact of conventional and second-generation PD fluids is described; novel strategies and innovative PD fluid types are discussed.

## Peritoneal Membrane Anatomy and Physiology

**Figure 1:**
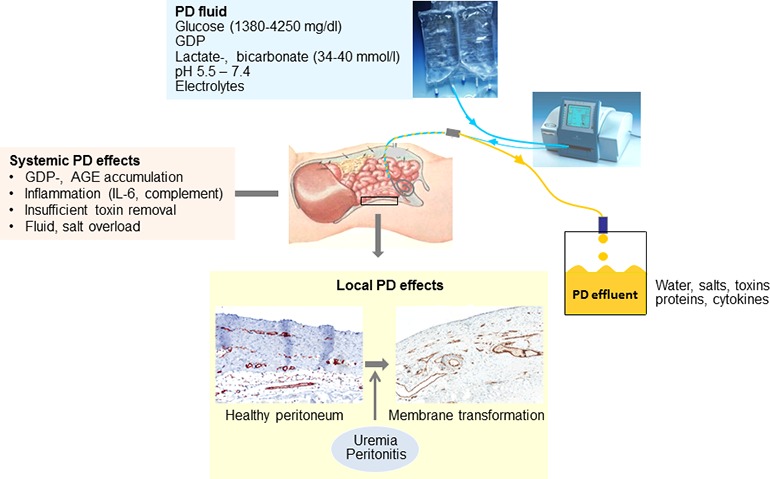
Scheme of peritoneal dialysis application, peritoneal dialysis (PD) fluid composition, and local as well as systemic effects. Three vessel layer structure of the healthy peritoneal membrane as published previously (Schaefer et al., 2016a).

The peritoneum is a delicate structure covering the entire peritoneal cavity. The parietal peritoneum is composed of a single layer of mesothelial cells and a submesothelial zone, which contains blood vessels, lymphatic vessels, and nerves that are mainly organized in a three layer structure (Blackburn and Stanton, 2014; Schaefer et al., 2016a) (Figure 1). The parietal peritoneal capillary density is age-dependent, with a two times higher blood vessel density in infants than in older children. In adults, peritoneal blood vessel density slowly increases with age but remains below the density observed in infants (Schaefer et al., 2016a). The age-dependent changes in peritoneal vascularization during childhood may be explained by the rapid increase in body dimension in early life, which should reduce a constant number of capillaries in an increasing tissue volume, as it is the case for the number of glomeruli found in a given cone kidney biopsy in children of different ages (Feneberg et al., 1998). Similar findings were observed for the lymphatic density, which overall is much lower than blood vessel density. Submesothelial thickness steadily increases until the age of 18 years and is again lower in adults. Respective percentile curves for age-appropriate evaluation of the peritoneum have been established (Schaefer et al., 2016a). The extracellular peritoneal matrix contains bundles of collagens and mucopolysaccharides and a small number of cells such as fibroblasts and mononuclear cells, including sparse CD45 lymphocytes and CD68 macrophages (Schaefer et al., 2016a).

The visceral peritoneum covers the abdominal organs and their supply structures, the mesentery, but no detailed systematic analyses across age groups have been performed. The omental peritoneum consists of a calretinine and podoplanin positive mesothelial cell layer as the parietal peritoneum. It covers the adjacent adipose tissue, which contains isolated bundles of large vessels and a much lower numbers of capillaries than the parietal submesothelium. The omental and parietal peritoneal blood capillary microvessel density are correlated. Thus, omental tissue specimen should be informative regarding the parietal peritoneal vessel density, at least in the non-diseased state (Schaefer et al., 2016a).

The peritoneum exerts numerous functions, many of which have already been recognized in the 19th century (Robinson, 1897; van Baal et al., 2017). The peritoneum maintains local homeostasis and provides protection from movement-induced frictions and adhesions by secretion of phospholipids, mainly phosphatidylcholine, together with surfactant proteins (SP-A, -B, -C) (Hills et al., 1998). In steady state, mesothelial cells produce 5–100 ml of peritoneal fluid containing complement factors (Tang et al., 2004; Zelek et al., 2016), immunoglobulins (Davies et al., 1990), defensins (Grupp et al., 2007), and immune cells like macrophages, lymphocytes, eosinophils, and mast cells (van Baal et al., 2017) that exert anti-infectious actions and regulate the inflammatory response (Isaza-Restrepo et al., 2018). *In vitro*, mesothelial cells migrate in an AQP-1-dependent manner (Ryu et al., 2012; Zhai et al., 2012), suggesting efficient wound healing capacity of superficial peritoneal erosions. Tissue remodeling is balanced by profibrotic cytokines and tissue inhibitors of metalloproteinase and by extracellular matrix degrading proteins such as metalloproteinases, gelatinase, and collagenase (Marshall et al., 1993; Ma et al., 1999). Plasminogen activator is responsible for a physiological fibrinolytic activity of peritoneum, and reduced concentrations following abdominal surgery promote adhesion formation (Holmdahl et al., 1996).

Angiogenesis in postnatal development is controlled by cytokines, including vascular endothelial growth factor (VEGF) and angiopoietins, and their receptors (Eklund and Saharinen, 2013). Angiogenesis is tightly regulated through a balance between activating and inhibiting signals (Fagiani and Christofori, 2013). Adult vasculature is quiescent, but blood vessels retain a high plasticity in order to respond to angiogenic signals after inflammation or injury. These angiogenic mechanisms should also be active in the peritoneum.

The omental fat tissue (Summers, 2006) generates numerous hormones and cytokines involved in immune responses and angiogenic and neurogenic factors (Chamorro et al., 1993; Goldsmith, 2001). It is a lipid store and pools immune cells, and it can adhere to neighboring peritoneum to embank local inflammation (Hall et al., 1998).

Altogether, the peritoneum is of clinical impact in various conditions such as postoperative adhesions (Hills et al., 1998; Arung et al., 2011), in patients with abdominal and gynecological carcinoma (Lemoine et al., 2016), and in patients with chronic kidney disease stage 5D requiring dialysis (CKD5D). This review focuses on the usage of the semipermeable peritoneum as a biological dialysis membrane, its transformation with peritoneal dialysis (PD), and current concepts and future prospects to improve PD efficacy and sustainability.

## Peritoneal Dialysis

PD is a life-saving, renal replacement therapy for a worldwide increasing number of patients with CKD5D. PD removes excess water and electrolytes as well as metabolic waste products by osmosis across a concentration gradient between the blood and the PD fluid and ultrafiltration-associated solvent drag (convection). PD is a cost-effective, home-based therapy and has significant advantages over hemodialysis (HD), in particular, regarding quality of life. Early patient outcome is at least similar to patients on HD (McDonald et al., 2009; Waldum-Grevbo et al., 2015). Despite these benefits, only a small number of dialysis patients receive PD, in Europe about 13% and in the USA about 10% (Mehrotra et al., 2016; Kramer et al., 2018). This discrepancy is in part explained by major limitations of PD. Infectious complications, mainly peritonitis, and the PD fluid induced progressive deterioration of the PD membrane with chronic PD, lead to PD function deterioration and eventually technique failure. As with hemodialysis, the uremic toxin and water removal capacity is far below physiological renal function. Most of the patients require strict dietary control and pharmacological treatment, such as phosphate binders, but the vast majority of patients are salt, fluid, and toxin overloaded. In particular, dietary phosphate and sodium intake are inadequately compensated by PD and essentially contribute to high blood pressure (Ortega and Materson, 2011), CKD mineral bone disorder, and cardiovascular disease (CVD). Mortality rates of both hemodialysis and PD patients are 40-fold higher compared to the age-related healthy population; accelerated cardiovascular disease is the primary cause of death (de Jager et al., 2009).

## PD Fluid Composition

The osmotic agent most frequently applied is glucose. PD fluids contain glucose at concentrations of 10- to 50-fold above physiological serum concentrations. Glucose creates an osmotic gradient with an osmolality of about 50–150 mOsmol/l above serum osmolality allowing for removal of water (called “ultrafiltration”) and of electrolytes and toxins by ultrafiltration-associated convection. Correction of metabolic acidosis is achieved by uptake of a buffer compound, lactate or bicarbonate, present in the dialysate at concentrations of 34–40 mmol/l.

First-generation PD fluids contain 35–40 mM lactate buffer and have an acidic pH of 5.5. The low pH aggravates the detrimental effects of the high lactate concentrations on peritoneal mesothelial and leukocyte function (Topley et al., 1988, 1996). During heat-sterilization and prolonged storage, high amounts of glucose degradation products (GDP) are formed, e.g., methylglyoxal and 3,4-dideoxyglucosone-3-ene. Peritoneal GDP exposure correlates with peritoneal advanced glycation end products (AGE) deposition and increasing peritoneal transporter status function, with the latter reflecting the PD fluid-induced peritoneal transformation process (Nataatmadja et al., 2018). GDP are rapidly absorbed into the circulation and increase systemic AGE concentrations (Zeier et al., 2003; Schmitt et al., 2007). AGE bind to the AGE receptor RAGE and trigger various intracellular events, such as oxidative stress and inflammation, leading to cardiovascular complications (Stinghen et al., 2016). Skin tissue AGE concentrations are increased in PD as compared to HD patients and independently associated with CV morbidity (Jiang et al., 2012).

To prevent GDP formation and to achieve a neutral to physiological pH, second-generation PD fluids have been introduced 20 years ago. These separate the buffer compound, lactate and/or bicarbonate, from the glucose, which is kept at a very low pH to reduce GDP formation during heat sterilization and storage. Prior to administration, the compartments are mixed; the final pH of the ready-to-use fluid is 7–7.4. Depending on the manufacturing process, GDP formation is substantially reduced but still varies considerably between different brands (Erixon et al., 2006). Second-generation PD fluids significantly reduce systemic GDP load and circulating AGE concentrations. The impact of 10–20% reduction in serum AGE concentrations achieved with low versus high GDP PD fluids on PD patient outcome, however, is still uncertain (Zeier et al., 2003; Schmitt et al., 2007).

PD fluids with an alternative osmotic agent contain icodextrin, a much less resorbed osmotic agent derived from starch. It allows for a slow but persistent colloid osmotic ultrafiltration and therefore can be used for a single long dwell per day (Dousdampanis et al., 2018; Morelle et al., 2018). Icodextrin fluid is especially applied in patients with high peritoneal solute transporter status and improves patient’s hydration status (Cho et al., 2013). Despite the absence of glucose and the very low GDP content, the acidic PD fluid has been associated with increased local and systemic inflammation (Parikova et al., 2003; Martikainen et al., 2005; Moriishi and Kawanishi, 2008; Velloso et al., 2014). Another alternative to glucose-based PD fluids is amino acid containing solutions, which are free of GDP and have an only slightly acidic pH of 6.7. For optimized nutrition of malnourished patients and to prevent increased serum nitrogen levels and metabolic acidosis (Dombros et al., 1990), they should be applied at a ratio of 1–4 with glucose-containing PD fluids (Tjiong et al., 2005). The nutritional effects are limited; stable isotope studies in adult CAPD patients yielded a 4% higher protein synthesis rate than patients treated with glucose-containing PD solution only (Tjiong et al., 2007). The biocompatibility of amino acid fluids remains uncertain, and experimental studies and findings in humans do not unanimously support the notion of improved peritoneal biocompatibility. Rats exposed to amino acid PD fluid had less peritoneal AGE deposition, lower VEGF levels, and a lower vessel density compared to rats treated with first-generation PD fluid (Mortier et al., 2004). *In vitro,* mesothelial cells exposed to amino acid PD fluid synthesized less HSP72, released more IL-6 and prostaglandin E2, and had superior viability as compared to acidic, high GDP fluid (Bender et al., 2008). Others, however, reported more mesothelial nitric oxide (NO) synthesis (Reimann et al., 2004). NO plays a key signaling role in numerous biologic processes, including control of vascular tone and permeability, and angiogenesis, *via* an interaction with VEGF (Papapetropoulos et al., 1997). Human peritoneal endothelial NO synthase expression and activity increase with time on PD and are related to endothelial VEGF upregulation and peritoneal vessel density (Combet et al., 2000).

Altogether, limited progress has been achieved during the past 50 years of PD treatment regarding PD fluid technology and mainly consists of reduction of the GDP content, pH neutralization, introduction of the bicarbonate buffer and of two alternative osmotic compounds. Glucose-based PD fluids still predominate, and PD treatment still confers major local peritoneal and systemic toxicity (Figure 1) (Schmitt and Aufricht, 2016).

## Peritoneal Membrane Transformation with Chronic PD

In patients with CKD5, at the time of catheter insertion, the peritoneum already exhibits minor but distinct alterations, including submesothelial thickening and vasculopathy, as compared to controls with normal renal function (Williams et al., 2002). In diabetic patients, peritoneal changes at start of PD are even more pronounced and comprise mesothelial loss, mesothelial basement membrane thickening, vascular wall thickening, and inflammatory cell infiltration (Contreras-Velazquez et al., 2008). The latter and hypoalbuminemia are associated with technique failure and mortality rate. In pediatric CKD5 patients, an increase in parietal vessel density (Schaefer et al., 2018) was observed. In contrast, omental fat vessel density was found to be reduced in pediatric CKD5D, pointing to another distinct and early feature of CKD-related vascular disease (Burkhardt et al., 2016). Parietal peritoneal micromorphological changes are accompanied by vascular endothelial telomere shortening, mild inflammatory cell invasion, epithelial-to-mesenchymal transition (EMT), fibrin deposition, and TGF-β-induced SMAD phosphorylation (Schaefer et al., 2018). Compared to the subsequent PD-induced changes, morphological alterations are still mild and do not progress much in patients on HD (Williams et al., 2002).

In a landmark paper of Williams et al., severe transformation of the peritoneum was demonstrated with chronic PD in patients treated with acidic, high GDP fluids (Williams et al., 2002). These changes included progressive loss of the mesothelial cell layer, a massive increase in submesothelial thickness especially in patients with more than 4 years of PD, and rapidly progressing, severe peritoneal vasculopathy. Number of peritoneal vessels per peritoneal section length was increased at the time of PD-related surgery and in patients with PD membrane failure, i.e., insufficient peritoneal transport function, as compared to a small group of patients with normal renal function. The study group did not relate their histologic findings to PD function and patient outcome; however, resulting therapeutic complications of long-term PD have repeatedly been described. Peritoneal solute transport gradually increases with time on PD, particularly when increasing concentrations of glucose are applied (Davies et al., 1998, 2001). Ultrafiltration capacity declines and eventually results in long-term ultrafiltration failure, which is often characterized by impaired osmotic conductance to glucose and reduced free water transport (Krediet and Struijk, 2013). High solute transport predicts technique failure and is associated with poorer patient survival (Davies et al., 1998). Peritoneal protein clearance also increases during the course of PD, but to a relatively smaller extend (Struijk et al., 1991; Ho-dac-Pannekeet et al., 1997).

Introduction of neutral pH, low GDP fluids raised hope to prevent long-term deterioration of the peritoneal membrane, based on numerous *in vitro* and experimental *in vivo* studies. These studies suggested improved local host defense (Mortier et al., 2003), reduced mesothelial damage (Grossin et al., 2006) and EMT (Bajo et al., 2011), less peritoneal GDP and AGE deposition, less TGF-β and VEGF signaling, and less submesothelial fibrosis and angiogenesis, altogether resulting in better preservation of peritoneal ultrafiltration capacity (Mortier et al., 2004, 2005; Rippe, 2009). Respective clinical trials were less consistent. Compared to first-generation PD fluids, administration of neutral pH, low GDP fluids resulted in higher CA125 effluent concentrations (Haas et al., 2003; Szeto et al., 2007), a putative marker of mesothelial cell viability and lower hyaluronic acid and procollagen peptide concentrations, suggesting improved peritoneal membrane integrity (Williams et al., 2004). A declining incidence of encapsulating peritoneal sclerosis has been associated with low GDP fluid usage (Nakao et al., 2017). Residual renal function, a major predictor of patient outcome, was better preserved (Kim et al., 2008; Haag-Weber et al., 2010; Johnson et al., 2012b). While superior residual renal function during the first year of PD may be related to less-effective fluid removal and consequent volume expansion with neutral pH, low GDP fluid, the long-term effect could be related to lower renal GDP and AGE exposure (Cho et al., 2014; Yohanna et al., 2015). The Euro-Balance trial, a randomized, two times 12-week crossover trial, demonstrated improved residual renal function together with decreased peritoneal ultrafiltration with the pH neutral, low GDP fluid, as compared to the first-generation, acidic high GDP solution (Williams et al., 2004). The largest study up to now, the BalANZ trial yielded a lower risk of anuria and lower ultrafiltration and higher solute clearance rates with the low GDP fluid during the first 9 months of PD. Over the entire 2 study years, the increase in solute transport and ultrafiltration decline were less pronounced with the low GDP fluid, resulting in comparable peritoneal membrane function at the study end with either fluid (Johnson et al., 2012b). A recent meta-analysis confirmed that neutral pH, low-GDP solutions result in a higher D/P creatinine during the first 6 months of treatment as compared to acidic, high GDP fluids but not subsequently (Yohanna et al., 2015). Peritonitis incidence and severity were reduced in the BalANZ trial and in another randomized, parallel trial over 2 years (Johnson et al., 2012a; Farhat et al., 2017), whereas other randomized trials did not report such differences (Williams et al., 2004; Szeto et al., 2007). Of note, these studies all compared the neutral pH, lactate-buffered, low GDP fluid with the acidic, lactate-buffered, high GDP PD fluid. Consecutive 1-day and 12-week randomized crossover studies in children comparing physiological pH, pure bicarbonate-buffered, low GDP fluid with first-generation PD fluid demonstrated similar ultrafiltration rates and a similar to 10% lower small solute transport rate with the former, which is in contrast to the reduced ultrafiltration and increased solute transport rates reported with neutral pH, lactate-based, low GDP fluids (Schmitt et al., 2002; Haas et al., 2003).

**Figure 2:**
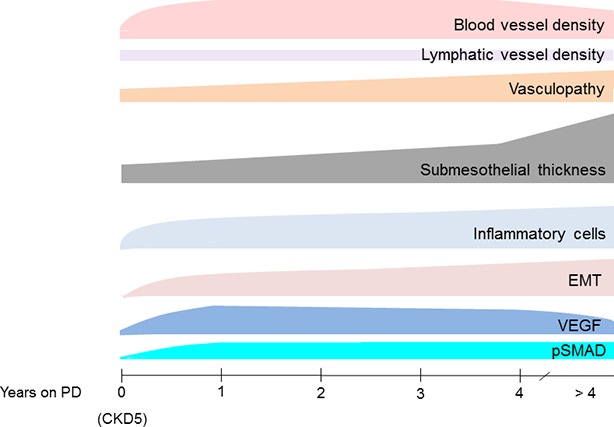
Transformation of the peritoneum with time on PD treatment with neutral pH, low GDP PD fluids. Blood microvessel density substantially increases within few months of PD. It is closely correlated with endothelial surface area, which presents the primary barrier for transport across the peritoneal dialysis membrane. Percentage of patients with substantial inflammatory cell infiltration and EMT increases with time on PD. VEGF signaling is particularly induced within the first year of PD, the TGF-β signaling cascade (pSMAD) activation is delayed but remains high during long-term treatment (Schaefer et al., 2018).

Peritoneal biopsies are usually not performed within clinical routine and limited to occasion of abdominal surgery required for other reasons. On the other hand, they are well tolerated, even in small children, and highly informative. They provide information not only on acute inflammatory but also on chronic PD-induced peritoneal damage and should allow for a prognostic estimate of PD performance. Scientific impact of peritoneal tissue analysis is considerable. Based on findings in 100 patients with diseases not affecting the peritoneal integrity and 90 CKD5 patients at time of first PD catheter insertion, Schaefer et al. (2018) analyzed 82 children on PD with low GDP fluids and revealed unexpected findings. In patients with a median PD vintage of 4 months, peritoneal blood capillary density and number per section length doubled, endothelial exchange area increased, and the three-layer structure has turned to a rather homogenous vessel distribution. Hypervascularization further increased in the majority of patients after 9 months of PD and remained largely unchanged thereafter. Peritoneal vessel density independently predicted glucose and creatinine transport. Vasculopathy, already present at time of PD initiation significantly progressed. In contrast, lymphatic vessel density remained largely unchanged in all PD patient groups. Submesothelial thickening progressed slowly and was severe in patients on PD for more than 4 years. These changes were accompanied by induction of VEGF- and TGF-β-induced SMAD phosphorylation, by EMT and inflammatory cells invasion (Schaefer et al., 2018) (Figure 2). This first study looking in detail in a larger number of patients into early and long term induced peritoneal changes and applying digital imaging analysis suggests that the assumption of significantly improved biocompatibility with neutral pH, low GDP fluids cannot be maintained (Blake, 2018). Still, conclusions need to be drawn with caution, and comparison with high GPD fluids is difficult. Children are uniquely suited for the analysis of specific CKD- and PD-related pathomechanisms, since, different from adults, they mostly suffer from underlying diseases not affecting the peritoneum (Harambat et al., 2012) and they are largely free of lifestyle and aging-related tissue damage. On the other hand, findings cannot necessarily be transferred altogether to the adult PD population. Angiogenesis may be regulated differently in growing children, and factors absent in children may have an impact on peritoneal pathomechanisms in elderly PD patients. Neutral pH, low GDP fluids have been recommended by the European Pediatric Dialysis Working Group in 2011 (Schmitt et al., 2011), and the majority of European children are now treated with low GDP fluids. PD vintage and body surface area adjusted dialytic glucose exposure matched comparison with high GDP fluid treatment thus far has been limited to a total of 30 children. After 1 year of PD, children on high GDP PD had a higher degree of vasculopathy and more submesothelial thickening (Schaefer et al., 2016b). Similar findings were reported in 24 adult Japanese patients on PD for about 4.5 years. Peritoneal AGE accumulation, submesothelial thickening, and vasculopathy were less severe with low GDP usage (Kawanishi et al., 2013). In a subsequent study from the same group including additional patients, the protective effect of low GDP fluid on vasculopathy was reconfirmed (Hamada et al., 2015). Del Peso et al. (2016) compared 23 low and 23 high GDP PD-treated patients matched for PD vintage, and the mean treatment duration was 2 years. The mesothelial cell layer was better preserved, and vasculopathy was less pronounced in the patients on low GDP PD. In children, better preservation of the mesothelium cell layer could not be demonstrated, possibly due to the fragility of the pediatric samples and related processing artifacts. Altogether, these findings suggest distinct benefits of second over first-generation PD fluids, but higher patient numbers are needed to draw firm conclusions, at best in combination with functional data.

No peritoneal tissues have been obtained from patients on amino acids or icodextrin solutions, clinical trials, however, suggest better preservation of the peritoneal transporter status when icodextrin solution is added to glucose-based high GDP regime (Davies et al., 2005).

In order to improve PD fluid biocompatibility, an in-depth understanding of molecular mechanisms of PD-induced membrane transformation and of related systemic effects of PD is required. Derived surrogate biomarkers of PD-induced pathomechanisms may allow predicting individual PD patient prognosis at an early stage and to guide dialysis therapy and establish therapeutic interventions. Up to now in clinical practice, PD biomarkers are largely limited to effluent cell count and cell differential. A number of potential surrogate parameters of peritoneal pathophysiology are on the horizon but still far from being established in clinical routine (Aufricht et al., 2017).

## Molecular Mechanisms of PD-Induced Peritoneal Transformation

Progressive destruction of the mesothelial cell layer, angiogenesis, and fibrosis and ultimately (life-threatening) peritoneal sclerosis (EPS) are due to an array of molecular mechanisms, which interact with each other. Peritoneal vessel density predicts peritoneal solute transport and overshooting vessel formation reduces ultrafiltration capacity, unless major fibrosis has developed, which reduces the osmotic conductance of glucose (Krediet et al., 2000). Experimental and human biopsy studies not only related peritoneal VEGF synthesis to peritoneal angiogenesis (De Vriese et al., 2001; Schaefer et al., 2018) but also shedded light on further aspects of the angiogenic machinery. Monoclonal VEGF antibody bevacizumab inhibits peritoneal angiogenesis and fibrosis in response to chlorhexidine (Ada et al., 2015). TNP-470, an endothelial cell cycle, and tumor angiogenesis inhibitor decreased peritoneal VEGF expression, EMT, vessel density, and fibrosis (Yoshio et al., 2004). Administration of endothelin-1 receptor antagonists in mice attenuated PD-induced EMT, angiogenesis, fibrosis, and peritoneal functional decline (Busnadiego et al., 2015). Similar findings were obtained for endostatin, an endothelial cell proliferation and migration inhibitor, in a mouse model of EPS (Tanabe et al., 2007) and for intraperitoneal rho-kinase inhibition in a rat model of peritoneal fibrosis (Peng et al., 2013). Rapamycin decreased mesothelial cell VEGF synthesis and VEGF-C and VEGF-D release *in vitro*; combined PD and rapamycin treatment in mice reduced peritoneal EMT and thickening and submesothelial blood and lymphatic vessel proliferation as compared with mice exposed to PD fluid only (Gonzalez-Mateo et al., 2015). Addition of Tie2 fusion protein sTie2/Fc blocking Angiopoietin 2 downstream signaling to PD fluid infused once daily in uremic mice dose dependently reduces PD-induced peritoneal angiopoietin 2 synthesis and peritoneal hypervascularization (Xiao et al., 2013). Thus, several different interventions within the angiogenic signaling cascades can substantially reduce PD-induced peritoneal membrane transformation. Thus far, however, such approaches have not been tested in the clinical setting of PD.

While experimental studies clearly demonstrated reduction of peritoneal angiogenesis with low compared to high GDP fluids (Mortier et al., 2004, 2005), the role of the buffer compound is less clear. *In vitro*, bicarbonate-buffered low GDP fluid induced less endothelial tube formation than the respective lactate-based fluid, due to an increase in angiopoietin 1/2 ratio, that is, a shift towards vessel maturation, and tyrosine kinase receptor (TEK) translocation to the endothelial cell membrane, where it co-localized with vascular-endothelial cadherin, which stabilizes vessels (Eich et al., 2017). TEK plays a pivotal role in the regulation of sprouting and maturation of the vessels (Eklund and Saharinen, 2013). The finding was supported by a larger cross-sectional area of peritoneal vessels in eight bicarbonate fluid treated, peritonitis free children, as compared to the vessel area in age and glucose exposure matched children treated with the respective second-generation lactate PD fluid. Vessel size is an indicator of maturation (Suri et al., 1998). Up to now, only one, small size randomized trial comparing lactate and bicarbonate-buffered, neutral pH, low GDP fluids has been accomplished and – in line with the experimental findings – demonstrated better preservation of ultrafiltration achieved per gram of dialytic glucose exposure and body surface area in pediatric patients over 10 months with the bicarbonate fluid (Schmitt et al., 2013).

PD fluid toxicity induced early and pronounced peritoneal inflammation involving invasion of the PD membrane with macrophages and leucocytes, and inflammatory cytokine release is another major driver of structural and functional deterioration (Lambie et al., 2013, 2016; Schaefer et al., 2018). IL-6 is secreted by mesothelial cells after induction by IL-1ß and TNF-α (Topley et al., 1993). Individual differences in dialysate IL-6 concentrations have been linked to genetic polymorphisms (Siddique et al., 2015). *In vitro* and in mice, IL-6 was linked to VEGF production and thus angiogenesis *via* STAT3 and SP4 transcriptional factors (Catar et al., 2017); effluent IL-6 and VEGF concentrations are correlated (Pecoits-Filho et al., 2002). In PD patients, dialysate concentrations of the proinflammatory cytokine IL-6 are associated not only with higher peritoneal transporter status, i.e., faster solute and toxin removal, but also with ultrafiltration decline and protein loss (Lambie et al., 2013). In experimental PD, the anti-inflammatory Cox-2 inhibitor, celecoxib, reduced peritoneal inflammation, angiogenesis, and fibrosis and preserved peritoneal membrane function (Fabbrini et al., 2009).

Another key element of peritoneal membrane transformation is epithelial (mesothelial) to mesenchymal transition (EMT), i.e., migration of mesothelial cells into the submesothelium and transition to a myofibroblast cell type. Lineage tracing studies furthermore suggest that myofibroblasts may also be derived from type I collagen-producing submesothelial fibroblasts (Chen et al., 2014). EMT is triggered by profibrotic and inflammatory stimuli cytokines (Yanez-Mo et al., 2003; Margetts et al., 2005; Loureiro et al., 2011; Bowen et al., 2013). Myofibroblasts secrete inflammatory, proangiogenic, and profibrotic cytokines and extracellular matrix components (Aroeira et al., 2007). In CKD5 patients, only single isolated EMT cells are present in the submesothelium, but their numbers rapidly increase with PD (Schaefer et al., 2018). In multivariate analysis, peritoneal EMT was independently associated with submesothelial thickness and with the microvessel number per mm tissue section. In experimental PD, EMT and associated peritoneal membrane damage can be inhibited by intraperitoneal BMP-7, antagonizing TGF-β signaling (Loureiro et al., 2010). TGF-β signaling again is centrally involved in the peritoneal fibrotic process as shown in various animal models of PD (Margetts et al., 2001) and in humans (Zhou et al., 2016; Schaefer et al., 2018). TGF-β is secreted by resident (myo-) fibroblasts, with different fibroblast subgroups having different profibrotic properties. Glycopeptide Thy1-positive fibroblasts exhibit particular profibrotic and myofibroblast features (Kawka et al., 2017). MicroRNA (miR) array studies identified miR-21 and miR-31 to be highly expressed and induced by TGF-β in mesothelial cells and to correlate with mesenchymal transition *in vitro*. Micro ribonucleic acid-21 and miR-31 are upregulated in the peritoneum of PD patients, and their effluent concentrations are associated with icodextrin and low GDP fluid use and related to peritonitis count and effluent IFN-γ concentration. Altogether these findings suggest a great potential of these miRs as biomarker for membrane change in patients receiving PD (Lopez-Anton et al., 2017), respective large size clinical trials are needed.

## Systemic Impact of PD Fluid Bioincompatibility

Rather than mitigating CKD-associated pathomechanisms, such as inflammatory, carbonyl, and oxidative stress, PD, while partially replacing renal function, adds additional risk factors. CKD-associated vasculopathy, prevalent even in young CKD patients, is further accelerated by PD. Potential pathomechanisms include the peritoneal glucose uptake, the additional GDP, and consequent AGE load and PD-associated inflammation. In a cohort of almost 1,000 PD patients, intraperitoneal inflammation was the most important determinant of peritoneal solute transport but did not affect patient survival (Lambie et al., 2013). In contrast, systemic inflammation associated with comorbidity and independently predicted patient survival, suggesting independent peritoneal and systemic processes being active. Other studies point to a strong link between PD treatment and vasculopathy. Whole exome expression analyses of omental arterioles isolated from children with normal renal function, with CKD5D and while on low GDP PD revealed activation of metabolic processes in CKD5D arterioles and of inflammatory, immunologic, and stress-response cascades in arterioles of PD patients. The latter exhibited particular upregulation of the complement system and respective regulatory pathways, with concordant findings at the proteomic level. In independent validation cohorts, PD specimens had the highest abundance of omental and parietal arteriolar C1q, C3d, terminal complement complex and of phosphorylated SMAD2/3, a downstream effector of TGF-β. Furthermore, in the PD parietal arterioles, C1q and terminal complement complex abundance correlated with the level of dialytic glucose exposure, the abundance of phosphorylated SMAD2/3, and the degree of vasculopathy (Bartosova et al., 2018). The close correlation of vascular TGF-β-induced SMAD2/3 phosphorylation and the severity of vasculopathy is supported by recent genome wide association, and systems biology studies identified the TGF-β–SMAD pathway to be strongly associated with coronary artery disease (Zeng et al., 2016). The analysis of small arteries and precapillary arterioles at least 1 mm below the mesothelial surface and thus beyond the PD penetration level (Stachowska-Pietka et al., 2012) is of particular interest because they control peripheral resistance and microcirculation. Vasculopathy in this part of the arterial tree predicts left ventricular hypertrophy and cardiovascular events in hypertensive patients (Rizzoni et al., 2003; De Ciuceis et al., 2007). Concentrations of effluent complement protein have been linked to overall mortality in PD patients (Zelek et al., 2016).

## Novel PD Fluid Prototypes

Severe peritoneal damage still observed with low GDP fluids suggests that glucose per se has a major detrimental effect and that glucose sparing should mitigate PD-associated sequelae. Adding icodextrin and amino acid solution to a glucose-based PD regime improved glycated hemoglobin and lipid profile as compared to the glucose only PD regime, but deaths and serious adverse events, including several related to extracellular fluid volume expansion, have been reported to increase in the intervention group (Li et al., 2013). PD fluids with lower sodium concentration increased sodium removal and improved blood pressure but accelerated residual renal function decline (Blake, 2016; Rutkowski et al., 2016). In a small size crossover trial in adults and a pilot study in children, adapted automated PD, i.e., combining sequential short- and longer-dwell exchanges, with small and large dwell volumes, resulted in higher solute and fluid removal as compared to the standard regime with comparable dialysate fluid turn over and dialysis time (Fischbach et al., 2016). This concept awaits validation in extended clinical trials.

Replacing glucose by novel osmotic agents is a promising way to go in order to improve PD fluid biocompatibility. About 3.5% taurine-based PD fluid achieved equivalent ultrafiltration as glucose-based PD fluid and induced less mesothelial and fibroblast-like cell proliferation in rats (Nishimura et al., 2009). Hyperbranched polyglycerol containing PD fluid achieved similar solute and water transport rates in rats and induced less peritoneal membrane damage (Mendelson et al., 2013; Du et al., 2016), but data on the metabolism of polyglycerol in plasma and ramifications of plasma accumulation and tissue disposition with long-term use are scant. A recent study in obese type 2 diabetic ZSF1 rats over 3 months suggests less systemic adverse effects on the kidneys and the plasma oxidative status with hyperbranched polyglycerol fluid as compared to second-generation and icodextrin PD fluid (La Han et al., 2018).

**Figure 3:**
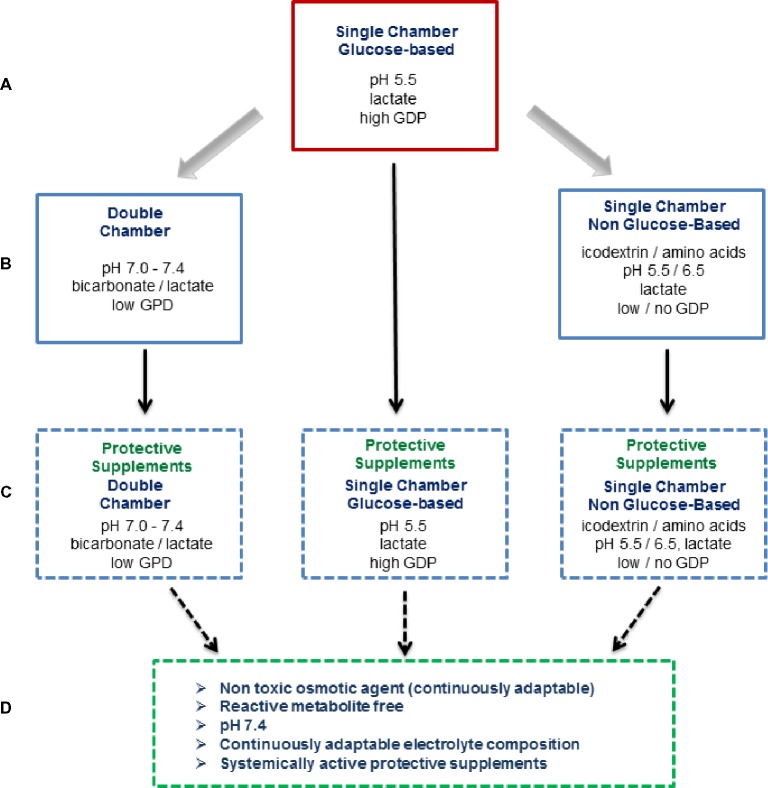
Overview on currently applied and potential novel PD fluid types. For about 50 years, conventional PD fluids have been based on glucose as the osmotically active agent and are heat sterilized in single-chamber bags at acidic pH together with selected electrolytes (Na, Ca, Mg, and Cl) and a buffer (lactate), which results in major glucose degradation product (GDP) formation **(A)**. In the 1990s, second-generation PD fluids were developed. These multi-chamber bag systems substantially reduce GDP generation and allow for a physiologic buffer compound (bicarbonate) and a neutral pH of the ready-to-use PD fluid. At the same time, alternative osmotic compounds were introduced, an amino acid mixture and the oncotically active glucose polymer icodextrin **(B)**. At present, protective agents counteracting local and systemic PD fluid toxicity are being developed, with alanyl-glutamine supplemented PD fluids have shown promising effects in first clinical trials **(C)**. The fourth generation PD fluid type depicted reflects the vision of the ultimate future PD fluid **(D)**.

A different approach to more biocompatibility PD fluids is addition of protective compounds counteracting peritoneal fluid toxicity (Figure 3). PD fluids result in cellular stress and also suppress the natural stress response mechanisms, e.g., exerted by heat shock proteins (HSP) (Macario and Conway de Macario, 2007). Glutamine, a non-essential amino acid, has been shown to restore the cellular stress response pathway HSP27/72, which is suppressed by PD fluids. Addition of the dipeptide alanyl-glutamine to first- and second-generation PD fluid improved mesothelial cell stress response and cell survival *in vitro* and *in vivo* (Kratochwill et al., 2012). In uremic rat and mouse models of PD, alanyl-glutamine reduced peritoneal thickness, and angiogenesis, and peritoneal αSMA, IL-17, TGF-β, and IL-6 (Ferrantelli et al., 2016). In a first clinical trial, effluent cell HSP72 expression was increased following a 4-h dwell with alanyl-glutamine supplemented first-generation PD fluid, and the effluent increased TNF-alpha release from LPS-stimulated peripheral blood mononuclear cells as compared to non-supplemented PD fluid. In post peritonitis patients, IL-6 and IL-8 effluent concentrations were reduced (Kratochwill et al., 2016). In a subsequent randomized crossover study, 41 patients were treated with alanyl-glutamine supplemented second-generation PD fluid over a period of 8 weeks each. Intraperitoneal alanyl-glutamine increased CA-125 appearance rate and effluent cell LPS-stimulated IL-6 release, and the peritoneal transport of uric acid, phosphate, and potassium was higher and the peritoneal protein loss was reduced (Vychytil et al., 2018). These studies demonstrate significant benefits of alanyl-glutamine enriched PD fluid on peritoneal membrane integrity, immune competence, and transport function. A phase 3 trial is now needed to translate these encouraging effects into hard clinical outcomes.

Experimental PD studies and a clinical pilot study in 4 patients suggest good tolerability of carnitine supplemented PD fluids and superior ultrafiltration than achieved with 2.5% glucose solutions, despite lower osmolarity of the carnitine-containing solution (Bonomini et al., 2011). Addition of L-carnitine to acidic, glucose-based PD fluids in 27 non-diabetic patients improved insulin sensitivity assessed by euglycemic hyperinsulinemic clamp studies (Bonomini et al., 2013).

**Figure 4:**
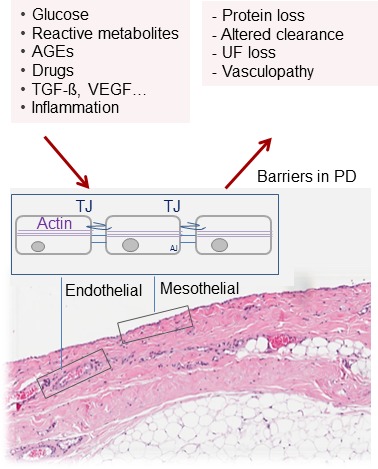
Barriers for solute and water transport in PD. The endothelial and mesothelial cell monolayers form leaky membranes. Intercellular junctions define the selective permeability properties and thus paracellular bulk flow of small and large solutes together with water. An in-depth understanding of these key elements of PD should provide promising therapeutic targets to improve PD efficacy, biocompatibility, and sustainability.

Understanding the molecular mechanism of peritoneal transport, its regulation by CKD and PD and pharmacological modification should be another way to improve PD biocompatibility and efficacy. Peritoneal membrane function has been well described by the three pore model (Rippe, 1993). Thus far, only the molecular basis of the “ultra-small pores” could be identified in mice, aquaporin-1 (AQP-1), which exerts 50% of water transport (Ni et al., 2006). The molecular counterparts of “small pores” and “large pores,” i.e., the mechanisms and regulatory machinery of the remaining 50% of the water transport, of solutes and size-dependent toxin removal are still unknown as are their modifications by uremia and PD. The primary transport barrier is the endothelium, and the role of the mesothelium is uncertain. Both capillary and mesothelial cell layers form leaky structures with similar *in vitro* transmembrane resistances but higher solute transport rates across the endothelial layer for 4–70 kDa dextrans (Horiuchi et al., 2009). Intercellular junctional complexes, including tight junctions, gap junctions, and desmosomes (Ito et al., 2000), define the selective permeability properties of the cell monolayer membrane and thus of bulk flow of small and large solutes together with water (Figure 4). Transcellular mechanisms imply transporters such as PiT for phosphate (Biber et al., 2013) and GLUT-1/2 and sodium glucose co-transporters such as SGLT-1/2 for glucose uptake (Debray-Garcia et al., 2016).

In-depth understanding of these key elements of peritoneal membrane transport function should provide promising therapeutic targets to improve PD efficacy, biocompatibility, and sustainability. Feasibility of this approach has been demonstrated for AQP-1. Dexamethasone twofold increased peritoneal AQP1 abundance and net ultrafiltration in rats without altering solute transport (Stoenoiu et al., 2003). AQP-1 agonist, AqF026, a chemical derivative of the aryl sulfonamide compound furosemide, increased water transport after 60 and 120 min of dwell time by 15–20% (Yool et al., 2013). Although this strategy is promising to remove more water to achieve euvolemia, it does not increase salt removal and may aggravate thirst in patients. Still, these studies elegantly demonstrate that understanding and modulating the peritoneal water, salt, and toxin transport mechanisms are a promising area of research, hopefully resulting in major improvement of PD patient outcome.

## Résumé

The recent findings on histomorphological alterations of the peritoneum with so-called biocompatible, neutral pH PD fluids are disappointing and raised the question whether current concepts of PD fluid biocompatibility are “dead” (Blake, 2018). These sobering findings, however, have to be balanced against patient-related outcome parameters. There is an early survival advantage for PD as compared to HD, together with advantages of quality of life and autonomy to this home-based, cost-effective therapy. A recent analysis of the ERA-EDTA registry, which comprehensively collects real-life data from European countries, suggests an increasing 5-year patient survival benefit of PD over HD over the last 20 years (van de Luijtgaarden et al., 2016). In Europe, low GDP fluids have been licensed about 20 years ago and have increasingly been applied since then. At present, it is unclear that which factors contribute to these encouraging trends, but it is tempting to speculate that the lower systemic GDP and AGE load associated with low GDP PD fluid use (Zeier et al., 2003; Schmitt et al., 2007), an improved local host immune defense system possibly resulting in less frequent and less severe episodes of peritonitis (Johnson et al., 2012c), and the better preservation of residual renal function (Cho et al., 2014) play a significant role. Further large-scale patient-related analyses are needed to delineate the specific PD-related risk factors and potential countermeasures, as well as the development of novel PD fluid types, which not only mitigate peritoneal damage but also systemic sequelae of chronic PD.

## Author Contributions

MB and CS performed the literature search and wrote the manuscript. Both authors approved the final version of the manuscripts.

### Conflict of Interest Statement

CPS has obtained lecturing honoraria, travel support, and investigator-initiated research funding from Fresenius Medical care and lecturing and consulting honoraria from Baxter.

The remaining author declares that the research was conducted in the absence of any commercial or financial relationships that could be construed as a potential conflict of interest.

The handling editor declared a past collaboration with the authors.
